# Modeling and Characterization of the Implant Intra-Body Communication Based on Capacitive Coupling Using a Transfer Function Method

**DOI:** 10.3390/s140101740

**Published:** 2014-01-20

**Authors:** Kai Zhang, Qun Hao, Yong Song, Jingwen Wang, Ruobing Huang, Yue Liu

**Affiliations:** School of Optoelectronics, Beijing Institute of Technology, Beijing 100081, China; E-Mails: 20040491@bit.edu.cn (K.Z.); wjw_bit@126.com (J.W.); huangruobing666@gmail.com (R.H.); Liuyue_11122@sina.com (Y.L.)

**Keywords:** implant intra-body communication, capacitive coupling, modeling, transfer function

## Abstract

Implantable devices have important applications in biomedical sensor networks used for biomedical monitoring, diagnosis and treatment, *etc.* In this paper, an implant intra-body communication (IBC) method based on capacitive coupling has been proposed, and the modeling and characterization of this kind of IBC has been investigated. Firstly, the transfer function of the implant IBC based on capacitive coupling was derived. Secondly, the corresponding parameters of the transfer function are discussed. Finally, both measurements and simulations based on the proposed transfer function were carried out, while some important conclusions have been achieved, which indicate that the achieved transfer function and conclusions are able to help to achieve an implant communication method with the highly desirable characteristics of low power consumption, high data rate, high transmission quality, *etc*.

## 1. Introduction

Intra-body communication (IBC) is a technology using the human body as transmission medium for electrical signals [1]. In general, IBC technology has two application forms: on-body IBC [2–4] and implant IBC [5,6], in which on-body IBC is used for the data exchange among electrical devices which are worn on the body [2,7], while implant IBC is used for the communication among implantable electrical devices [5].

**Figure 1.**
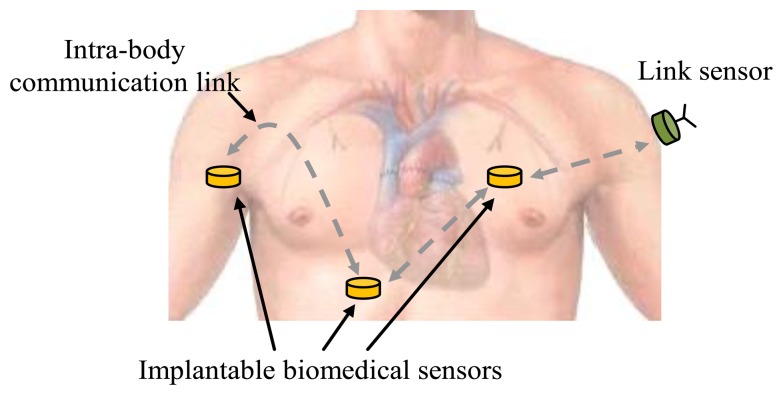
Implant communications based on IBC technology.

Like on-body IBC, the implant IBC provides benefits to many applications, such as biomedical monitoring systems [6,8] and other related application fields [9–11]. Compared with other implantable device communication methods [12,13], implant IBC has the advantages of low transmission power, small size, *etc.* [5]. Implant IBC can be applied in monitoring patient's condition and in the diagnosis and treatment of many diseases, including heart disease, neurological disorders and cancer detection [5,13,14], *etc*. In a biomedical monitoring system based on implant IBC, biomedical data are collected by implantable biomedical sensors located at different parts of the human body and are transmitted to other sensors using the IBC techniques, as shown in Figure 1. Finally, the data can be received and transmitted to the hospital by a link sensor, which is attached on the body, and integrated conventional wireless modules. Therefore, implant IBC is particularly important for implantable biomedical sensors to communicate with each other in biomedical monitoring system.

However, the previous works in this field have some limitations, which can be summarized as follows: (1) Comparatively higher signal attenuation. Previous investigations on implant IBC mainly concentrated on the implant IBC based on galvanic coupling [6,15], which has comparatively higher signal attenuation. In implant IBC based on galvanic coupling, an alternating current is applied with a pair of transmitter electrodes to the human tissue and detected by a pair of receiver electrodes [16,17]. Due to the fact that the two coupling electrodes of the transmitter contact with the body directly, a primary current flow between the coupler electrodes is established and only a small secondary current propagates into the conductive body parts [5]. As a result, the body effectively shorts the signal from the transmitter, which increases the signal attenuation and the power consumption, and greatly shortens the operation time of the implant [18,19]; (2) The lack of a corresponding mathematical model. As for the research of implant IBC, the corresponding mathematical model is very important for achieving the characteristics of implant IBC [16,20]. However, the previous works failed to develop the corresponding transfer function of the implant IBC. As a result, some of the implant IBC phenomena can 't be explained in theory, while other characteristics remain unrevealed so far.

On the other hand, it has been proved that on-body IBC based on capacitive coupling has comparatively lower signal attenuation. In the on-body IBC based on capacitive coupling, only the signal electrodes of the transmitter and receiver are attached to the body skin directly, while both the transmitting ground electrode and the receiving ground electrode remain floating [10,21,22]. As a result, this avoids the body shorting the signal from the transmitter and more signal energy can reach the receiver electrodes [18]. Therefore, a comparatively lower power consumption can be achieved. However, the principle of IBC based on capacitive coupling has not been used in the implant IBC so far. In this paper, an implant intra-body communication method based on capacitive coupling has been proposed, while the modeling and characterization of this kind of IBC have been investigated.

The rest of the paper is organized as follows: In Section 2, a circuit model of the implant IBC based on capacitive coupling was developed, then the corresponding transfer function was derived. Some important parameters of the transfer function were discussed and modeled in detail in Section 3. In Section 4, measurement experiments were carried out for verifying the reliability of the proposed transfer function, while some important characteristics of the proposed method were studied. Finally, Section 5 concludes this paper.

## 2. Transfer Function

### 2.1. Circuit Model

**Figure 2.**
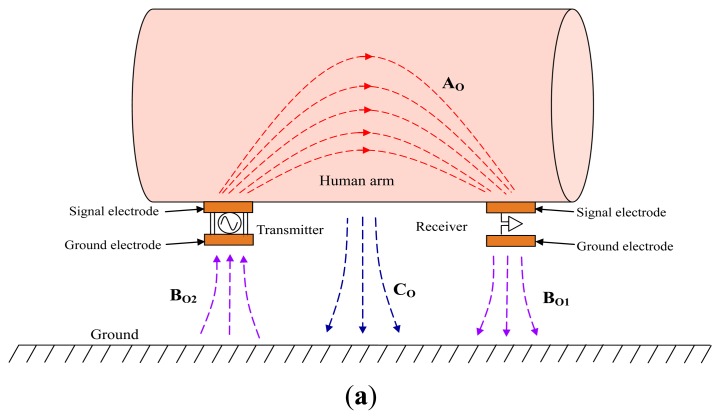

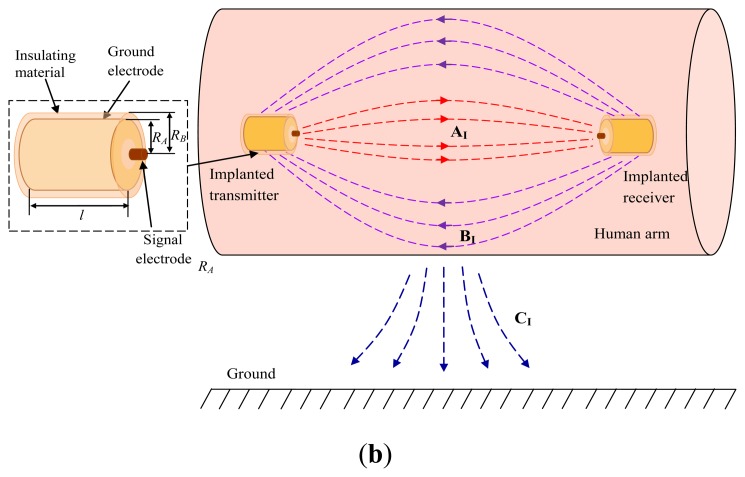
Schematic diagram of (**a**) on-body IBC based on capacitive coupling and (**b**) implant IBC based on capacitive coupling in the path of arm.

Firstly, the difference between the on-body IBC based on capacitive coupling and the implant IBC based on capacitive coupling is analyzed. In the on-body IBC based on capacitive coupling, as shown in Figure 2a, only two signal electrodes are attached on the human body (e.g., human arm), while an electric field **A_O_** forms between them though the body. Meanwhile, both the transmitting ground electrode and the receiving ground electrode remain floating, which results in an electric field **B_O2_** between the ground electrode of transmitter and ground as well as an electric field **B_O1_** between the ground electrode of receiver and the ground. Finally, the return path of signal is established by the electric field of **B_O1_**, the ground and the electric field of **B_O2_**, thereby signal transmission between the transmitter and the receiver can be achieved. On the other hand, there is also an electric field **C_O_** between the body and the ground because of the body potential, which affects the signal transmission of IBC to some extent [1].

In the implant IBC based on capacitive coupling, both the transmitter and receiver are implanted into human body, as shown in Figure 2b. In our investigation, each implant capacitive electrode contains a signal electrode made of a metal stick and a ground electrode made of a metal cylindrical casing. Meanwhile, the signal electrode contacts with the human tissue directly, and the ground electrode is insulated from the human tissue as well as the signal electrode by using an insulating shell, which avoids the body shorting the signal between the signal electrode and the ground electrode. Moreover, compared with the coupling between the signal electrode and the ground electrode, a comparatively bigger capacitive coupling between the two ground electrodes can also be achieved because of the comparatively bigger surface area of the two ground electrodes. Thereby, signal transmission between the transmitter and the receiver can be achieved with low attenuation.

In the implant IBC based on capacitive coupling shown in Figure 2b, there is an electric field **A_I_** between the signal electrode of transmitter and that of the receiver in human tissue, which is similar to electric field **A_O_** shown in Figure 2a. On the other hand, instead of locating outside the human body as shown in Figure 2a, the return path of the implant IBC based on capacitive coupling locates inside the human body, which is the capacitive coupling represented as the electric field **B_I_** between the two ground electrodes through the insulating shell and human tissue. Therefore, signal transmission between the implanted transmitter and the implanted receiver can be achieved through electric field **A_I_** and **B_I_**. Additionally, there is also a coupling between the body and the external ground through the electric field **C_I_**, as shown in Figure 2b. Considering the fact that both the signal electrode and ground electrode couple with the ground through the human tissue, thereby the electric field **C_I_** affects the coupling between the ground electrodes (electric field **B_I_**) and that between the signal electrodes (electric field **A_I_**) synchronously.

**Figure 3.**
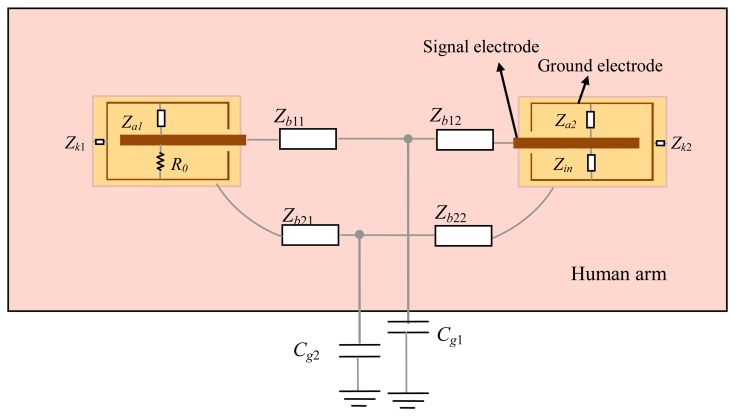
Circuit model of implant IBC based on capacitive coupling.

According to Figure 2b, the circuit model of the implant IBC can be obtained, as shown in Figure 3. The electrical model of each unit block can be represented as an impedance *Z*, which is equivalent to the parallel connection of corresponding capacitance *C* and resistance *R* [2,18], as shown in Equation (1):

(1) $$Z=\frac{1}{1/R+j\omega C}$$

In the circuit model of the transmitter, as shown in Figure 3, *R_0_* represents the output resistance of the transmitter, *Z_a_*_1_ is the impedance between the ground electrodes and the signal electrodes, and *Z_k_*_1_ represents the impedance of insulating shell between the ground electrode and the human tissue. On the other hand, *Z_b_*_11_ and *Z_b_*_12_ represent the transverse impedance between the two signal electrodes, and *Z_b_*_21_ and *Z_b2_*_2_ represent the impedance between the two ground electrodes. Meanwhile, the coupling capacitances between the human body and the external ground are represented as *C_g_*_1_ and *C_g_*_2_, which affect the coupling paths between the signal electrodes (*Z_b_*_11_ and *Z_b_*_12_) and that between the ground electrodes (*Z_b_*_21_ and *Z_b2_*_2_), respectively. Additionally, in the circuit model of the receiver, *Z_in_* represents the input impedance of the receiver, while the other parameters are similar to that of the transmitter.

**Figure 4.**
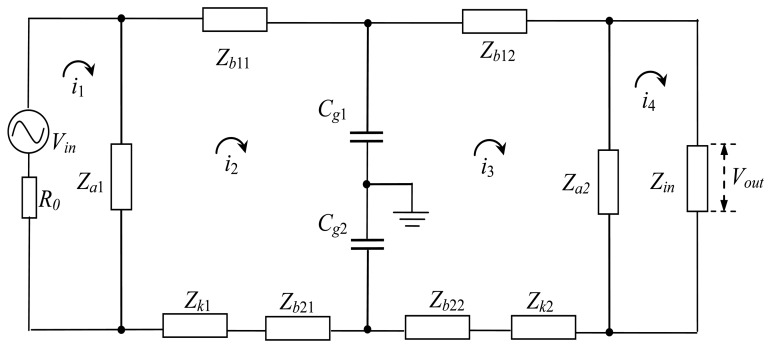
Equivalent circuit of implant IBC based on capacitive coupling.

The equivalent circuit of the circuit model in Figure 3 is shown in Figure 4, in which *V_in_* represents the output voltage of the transmitter, and *V_out_* represents the input voltage of the receiver.

### 2.2. Derivation of the Transfer Function

The transfer function of the implant IBC based on capacitive coupling can be derived by Kirchhoff voltage law (KVL) mesh equations [23,24], because the equivalent circuit of it is a linear system. In Figure 4, *i_n_* (*n* = 1, 2, 3, 4) is the current of the corresponding mesh, then the following equation can be expressed as:

(2) $$\left\{ \begin{array}{l} (R_{0}+Z_{a1})i_{1}-Z_{a1}i_{2}=V_{in}\\ (Z_{a1}+Z_{b11}+Z_{k1}+Z_{b21}+Z_{g1}+Z_{g2})i_{2}-Z_{a1}i_{1}-(Z_{g1}+Z_{g2})i_{3}=0\\ (Z_{a2}+Z_{b12}+Z_{k2}+Z_{b22}+Z_{g1}+Z_{g2})i_{3}-(Z_{g1}+Z_{g2})i_{2}-Z_{a2}i_{4}=0\\ (Z_{a2}+Z_{in})i_{4}-Z_{a2}i_{3}=0 \end{array}\right.$$

It is assumed that mesh impedance matrix **Z** contains the respective impedances in the circuit, which is a diagonal 4 × 4 square matrix as follows:

(3) $$\text{Z}=\left[\begin{array}{cccc} R_{0}+Z_{a1} & -Z_{a1} & 0 & 0\\ -Z_{a1} & Z_{a1}+Z_{b11}+Z_{k1}+Z_{b21}+Z_{g1}+Z_{g2} & -(Z_{g1}+Z_{g2}) & 0\\ 0 & -(Z_{g1}+Z_{g2}) & Z_{a2}+Z_{b12}+Z_{k2}+Z_{b22}+Z_{g1}+Z_{g2} & -Z_{a2}\\ 0 & 0 & -Z_{a2} & Z_{a2}+Z_{in} \end{array}\right]$$

On the other hand, the column matrix of the voltage sources **V** and the matrix of the mesh currents **I** can be expressed as:

(4) $$\begin{array}{cc} \text{I}=\left[\begin{array}{c} i_{1}\\ i_{2}\\ i_{3}\\ i_{4} \end{array}\right], & \text{V}=\left[\begin{array}{c} V_{in}\\ 0\\ 0\\ 0 \end{array}\right] \end{array}$$

As a result, Equation (2) can be summarized as a matrix equation:

(5) ZI=V

Furthermore, the mesh admittance matrix is determined by inverting the mesh impedance matrix as:

(6) $$\text{Y}={\text{Z}}^{-1}$$

Therefore, the current *i*_4_ can be obtained by calculating the mesh current matrix **I**:

(7) I=YV

Then the output voltage of the implant IBC can be expressed as:

(8) $$V_{\text{out}}=Z_{in}i_{4}$$

Finally, based on Equations (5) and (8), the attenuation of the signal transmission in the capacitive coupling IBC can be determined by:

(9) $$G=20\log_{10}(\frac{V_{\text{out}}}{V_{in}})$$

## 3. Parameters

The following is the discussion with respect to the parameters of the deduced transfer function.

### 3.1. Transverse Impedance (Z_b_)

Due to the fact that the human body generally consists of five layers (skin, fat, muscle, cortical bone, and bone marrow), *Z_b_* can be expressed as the parallel connection of the impedances corresponding to the different layers [25]:

(10) $$Z_{b}=\frac{1}{\sum_{n=1}^{5}\frac{1}{Z_{l}}}=\frac{1}{\sum_{n=1}^{5}\frac{1}{R_{n}}+\sum_{n=1}^{5}j\omega C_{n}}=\frac{L_{s}}{\sum_{n=1}^{5}\sigma_{nf}S_{n}+j\omega \varepsilon_{0}\sum_{n=1}^{5}\varepsilon_{\text{rnf}}S_{n}}$$

where *L_s_* is the length of the signal transmission path, *S_n_* is the cross-sectional area of the *n*th layer, *σ_nf_* and *ε_nf_* are the conductivity and the relative permittivity corresponding to the different layers and signal frequencies respectively which can be calculated from the Gabriel's results [26]. According to the Equation (10), the transverse impedances including the impedance between the two signal electrodes (*Z_b_*_11_, *Z_b_*_12_) and that between the two insulating shells (*Z_b_*_21_, *Z_b_*_22_) can be obtained.

### 3.2. Impedance of Insulating Shell (Z_k1_ and Z_k2_)

*Z_k_*_1_ and *Z_k_*_2_, which are the impedances of insulating shell between the ground electrode and the human tissue, can be obtained by Equation (1) using *C_k_*_1_, *C_k_*_2_ and *R_k_*_1_, *R_k_*_2_. The capacitances of *C_k_*_1_ and *C_k_*_2_ represent the capacitances between two coaxial cylinders, which are expressed by Equation (11):

(11) $$C_{k1}=C_{k2}=\frac{2\pi \varepsilon_{0}\varepsilon_{r}L}{\ln\frac{R_{B}}{R_{A}}}$$

where *ε_0_* is the permittivity of the vacuum, *ε_r_* is the relative permittivity of the insulating shell, *R_A_* is the radius of the ground electrode, *L* is the length of the ground electrode, and *R_B_* is the radius of the insulating shell, as shown in Figure 2b. On the other hand, *R_k_*_1_ and *R_k_*_2_ can be calculated by the equation *R* = *L*/*σA*, where *A* is the contacted area and *σ* is the conductivity of the insulating shell. Similarly, the impedance *Z_a_*_1_ and *Z_a_*_2_ can also be obtained by the above method.

### 3.3. Capacitance Between the Human Body and the External Ground (C_g1_, C_g2_)

**Figure 5.**
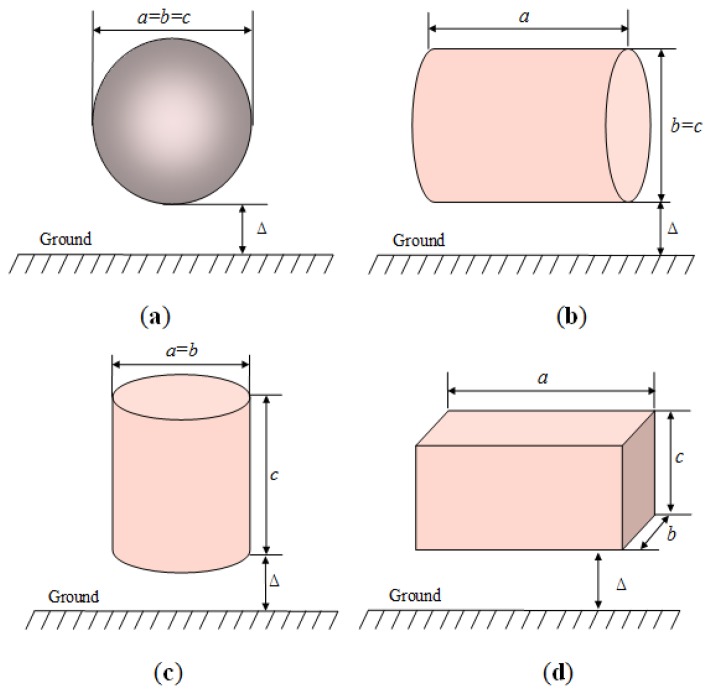
The parameters of *a*, *b* and *c* corresponding to the objects with different configurations, which include (**a**) sphere; (**b**) horizontal cylinders; (**c**) vertical cylinders; and (**d**) rectangular box.

It is assumed that if a person stands in an open space, and the human body is approximated as a conductive cylinder or sphere [1], then the capacitance (*C_g_*) between the human body and the external ground can be represented as [27]:

(12) $$C_{g}=C_{\infty }+C_{P}$$

where *C_∞_* is the capacitance of the object well above the ground and *C_p_* is the additional capacitance caused by the proximity effect of the ground. Generally, normalization is required to simplify the calculation of capacitance to the ground. In our investigation, *C_∞_* is derived from a parameter *l_e_*, which represents the equivalent length of the object and is defined as:

(13) $$l_{e}=(a+b+c)/3$$

where *a* is the length of horizontal direct, *b* is the width of horizontal direct and *c* is the height. The parameters of *a*, *b* and *c* corresponding to the objects with different configurations are shown in Figure 5 [27], which include sphere, horizontal and vertical cylinders, and a rectangular box. In addition, Δ is the smallest distance between the object and the ground.

As a result, the capacitance between the object and the ground of infinity *C_∞_*, can be assumed as a sphere with diameter *l_e_* in free space, which is calculated as follows [27]:

(14) $$C_{\infty }=2\pi \varepsilon_{0}l_{e}$$

In our investigation, the arm attached with the electrodes is abstracted as a cylinder, of which the diameter is *d* and the height is *l*. According to Equations (13) and (14), *C_∞_* between the arm and the ground of infinity can be calculated by:

(15) $$C_{\infty \_\text{arm}}=2\pi \varepsilon_{0}\left(2d+l\right)/3$$

On the other hand, the capacitance of *C_P_* can be approximated as [27]:

(16) $$C_{P}=\varepsilon_{0}\int_{S}\frac{dA}{h}$$

where *dA* is an elementary surface area, and *h* is the height above the ground of this area. Therefore, the additional capacitance (*C_P_arm_*) between the arm and the ground can be calculated by:

(17) $$C_{P\_\text{arm}}=\pi \varepsilon_{0}d[\frac{d}{4\Delta }+\ln(1+\frac{l}{\Delta })]$$

where Δ is the smallest distance between the arm and the ground.

## 4. Experiments and Discussion

In order to verify the validity of the proposed models and parameters, the measurements of implant IBC and the mathematical simulations based on the proposed transfer function were carried out. Moreover, the characteristics of the implant IBC based on capacitive coupling were also analyzed.

### 4.1. Experiment Setup

**Figure 6.**
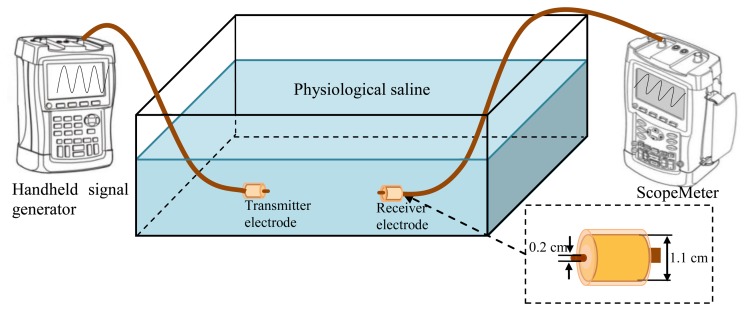
Measurement setup of implant IBC based on capacitive coupling.

In our investigation, the experiment setup of the implant IBC based on capacitive coupling was composed of a handheld signal generator, a ScopeMeter, a pair of implantable capacitive coupling electrodes and a rectangle tank, as shown in Figure 6. The handheld signal generator (DSO8060, *R*_0_ = 50 Ω) was used to provide the output signal at the transmitter terminal, and the ScopeMeter (Fluke 196C, *R_in_* = 1 MΩ and *C_in_* = 15 pF) was used to measure the signal at the receiving terminal. Both the handheld signal generator and the ScopeMeter were powered by battery for decreasing the influence of the external ground and simulating the actual application of implant IBC. Additionally, all the measurements were carried out at room temperature (298.15 K).

A rectangle tank with the size of 45 × 35 × 20 cm was used for simulating the human body, as shown in Figure 6. The tank was filled with physiological saline [28], which is assumed to be isotropic, as well as has the conductivity (*σ*) of 1.75 S/m and the relative permittivity (*ε_r_*) of 80.4, as shown in Table 1. Therefore, the resistance and capacitance of the transmission path can be obtained by *R* = *L*/*σA* and *C* = *ε_r_ε_0_A*/*L*, where *A* is the cross-section area of the transmission path, and *L* is the length of the transmission path. *C_∞_* of the measurement tank can be calculated by Equation (14), while its *C_P_* can be calculated according to Equation (16), which is equal to:

(18) $$C_{P\_\text{expriment}}=\varepsilon_{0}[\frac{ab}{\Delta }+2(a+b)\ln(1+\frac{c}{\Delta })]$$

In our experiment setup, the ground electrode of the capacitive coupling electrodes is cylindrical casing and packed with insulating shell (*σ* = 1 × 10^−14^, *ε_r_* = 3). The radius *R_A_* of the ground electrode is 0.55 cm, and the radius *R_B_* of the insulating shell is 0.6 cm. Meanwhile, the signal electrode with the radius of 0.1 cm, is contacted with the physiological saline directly, as shown in Figure 6.

**Figure 7.**
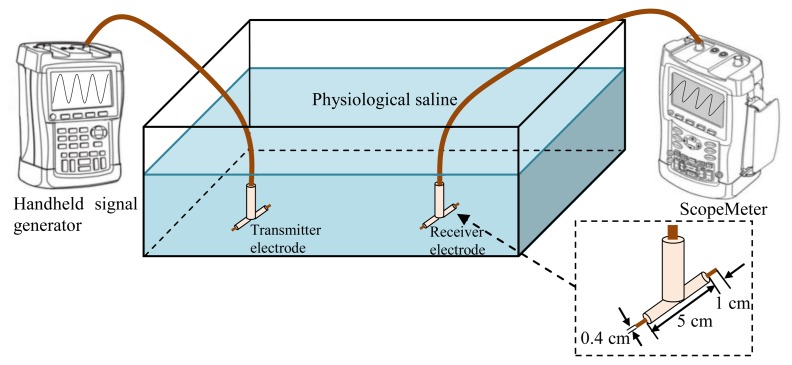
Measurement setup of implant IBC based on galvanic coupling.

Moreover, in order to verify the advantages of implant IBC based on capacitive coupling compared with the implant IBC based on galvanic coupling, the electrodes of the implant IBC based on galvanic coupling were also developed, which had two cylindrical copper endings (length 1 cm and diameter 4 mm) and the distance between them was 5 cm [5]. Figure 7 shows the experiment setup of the implant IBC based on galvanic coupling.

### 4.2. Comparison of Implant IBC Based on Two Coupling Methods

In this experiment, the separation distance between the transmitter electrode and the receiver electrode was set as 30 cm. Meanwhile, the sine wave signals with the amplitude of 4 V (peak-to-peak value) were applied on the transmitter electrodes. On the other hand, the signal frequency range of 100 kHz–40 MHz was chosen in our measurement, due to the fact that the power spectrum of the electrical signals produced by the biological processes mainly covers the low frequency range (less than 100 kHz) [5] and there is also the limitation of the circuit model in the high-frequency range [10].

**Figure 8.**
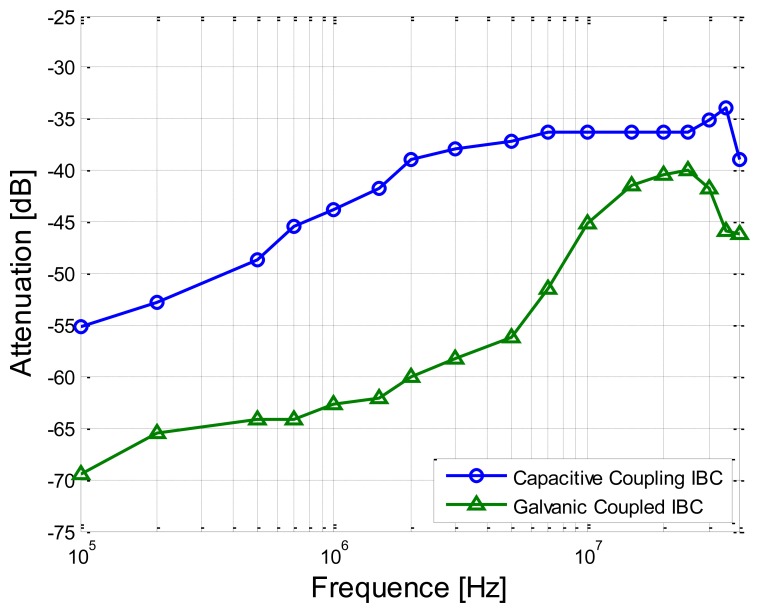
Measurement results of the proposed method and the implant IBC based on galvanic coupling.

Figure 8 shows the measurement results with respect to the frequency-dependent characteristics of the proposed method and the implant IBC based on galvanic coupling. We can find from Figure 8 that the attenuation of the proposed method is significantly lower (on average by 13.13 dB) than that of the implant IBC based on galvanic coupling. Meanwhile, both the two signal attenuation curves decrease gradually with the increasing of the signal frequency from 100 kHz to 2 MHz. However, the result of the IBC based on the galvanic coupling has comparatively bigger variation (the maximum deviation is up to 29.54 dB) in the frequency range of 2 MHz–40 MHz, while the result of the proposed method has comparatively smaller variation (the maximum deviation is only 3.90 dB) in the same frequency range. The above phenomenon can be explained that there is comparatively bigger coupling between the signal electrode and the ground electrode of transmitter in the IBC based on the galvanic coupling, thereby only lower signal energy can reach the receiver electrodes. On the contrary, in the proposed method, the mentioned coupling is weakened by using the insulating shell, thereby more signal energy can reach the receiver electrodes, which results in lower signal attenuation.

### 4.3. Verification of the Transfer Function

In order to verify the validity and the accuracy of the transfer function, both the measurements and the corresponding simulations with respect to the frequency-dependent characteristics of the proposed method were carried out under the conditions of the different signal transmission distances and heights.

**Figure 9.**
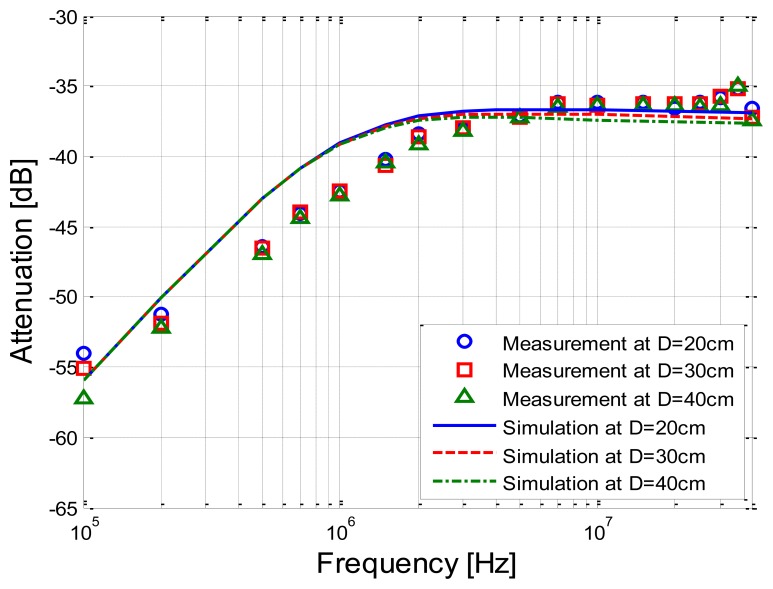
Comparison between measurements and simulation results corresponding to the different signal transmission distances.

Figure 9 shows the measurements and simulation results corresponding to the signal transmission distances of 20 cm, 30 cm and 40 cm, respectively. It can be seen from Figure 9 that the simulation results based on the developed transfer function basically coincide with the corresponding measurement results, while the deviations between the simulation and the measurement are limited within 3.93 dB. Meanwhile, both the simulation results and the measurement results decrease as the signal frequency increases from 100 kHz to 2 MHz, and have little variation within the frequency range of 2 MHz–40 MHz.

On the other hand, when the signal transmission distance increases from 20 cm to 40 cm, both the signal attenuations of the two results have little variation. For instance, an increase of 10 cm of the signal transmission distance only leads to an extra attenuation of 0.25 dB on average according to the measurement results. Similarly, the extra attenuation of the corresponding simulation is 0.14 dB on average, which indicates that both of them basically are not sensitive to the signal transmission distances.

**Figure 10.**
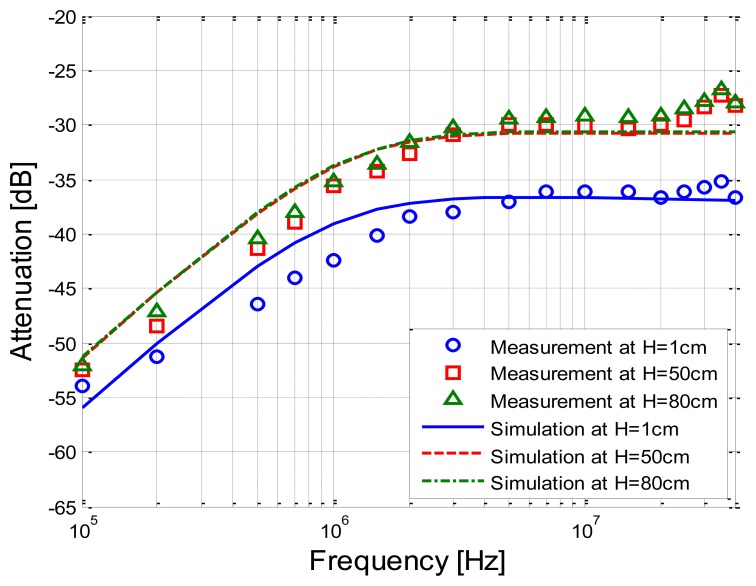
Comparison between the measurement results and simulation results corresponding to the different heights.

Moreover, under the condition that the height between the tank and the ground was set as 1 cm, 50 cm and 80 cm, respectively, while the signal transmission distance was set as 20 cm, the implant IBC experiments as well as the corresponding simulations based on the transfer function were carried out. Figure 10 shows the comparison between the measurement results and simulation results corresponding to the different heights.

It can be observed from Figure 10 that the mathematical simulation results also basically coincide with the corresponding measurement results. Meanwhile, the attenuation of the measurement results corresponding to the height of 50 cm is 6.00 dB less than that of the height of 1 cm on average, while a similar decrease (5.27 dB) can also be found in the simulation results. In addition, the curve of the measurement corresponding to the height of 80 cm overlaps with that of the height of 50 cm, and the corresponding variations are limited in 0.28 dB. Meanwhile, the similar phenomenon can be found in simulation results, and the variations are limited within 0.12 dB, which indicates that both the measurement and the simulation results remain basically unchanged as the height increases from 50 cm to 80 cm.

### 4.4. Characteristics of the Implant IBC Based on the Capacitive Coupling

In order to determine the characteristics of the proposed method, the corresponding simulations of the proposed method were carried out under the conditions of the different frequencies, signal transmission distances and heights based on the transfer function which has been verified. In our simulation, it is assumed that the human body is in static state at room temperature (298.15 K), which means that the capacitance to the ground keeps unchanged, and the influence of temperature variation is ignored [29,30].

#### 4.4.1. Characteristics of Frequency and Distance

**Figure 11.**
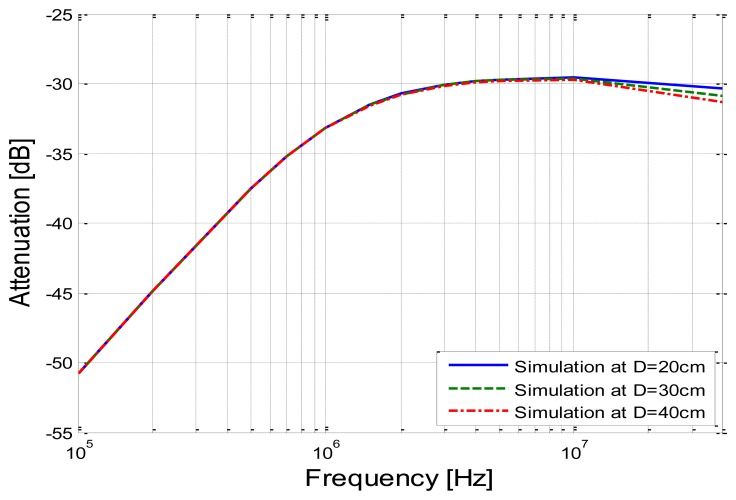
Simulation results corresponding to the different signal transmission distances and frequencies.

In this simulation, we assumed that the implantable capacitive coupling electrodes were embedded in the arm, of which the diameter was 10 cm. The attenuation curves corresponding to the different distances (20, 30 and 40 cm) are shown in Figure 11, in which the distance between the arm and the ground is 50 cm and the capacitance *C_g_*_1_ between the arm and the ground is equal to 7.99 pF.

It can be seen from Figure 11 that the attenuation becomes lower when the frequency increases from 100 kHz to 3 MHz, and it remains relatively stable when the frequency increases from 3 MHz to 10 MHz, which is similar to the results shown in Figure 9. On the other hand, the attenuation has a slight increasing when the frequency is higher than 10 MHz. Therefore, the comparatively lower signal attenuation can be achieved by using the proposed method in the case that the signal frequency range is within the range of 3 MHz–10 MHz. What's more, according to the results shown in Figure 11, the signal transmission distance has comparatively less effect on the signal attenuation. For instance, the mean deviation is only 0.06 dB in the frequency range of 100 kHz–40 MHz when the distance is increased by 10 cm. This phenomenon can be interpreted as that the impedance of the human body path (*Z_b_*) is much smaller than the other impedances of the return path (such as *Z_g_*_1_ and *Z_k_*_1_). As a result, the signal attenuation changes little with the increase of the transmission distance, which caused the increase of *Z_b_*.

#### 4.4.2. Characteristics of Height

**Figure 12.**
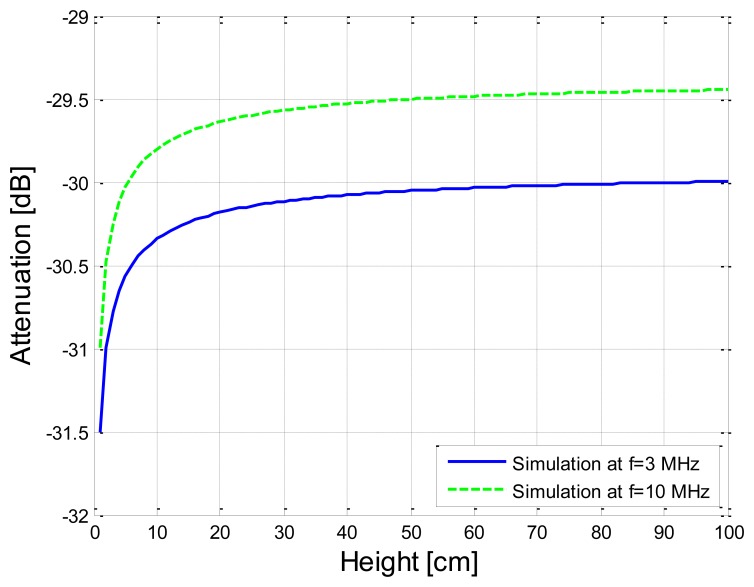
Simulation results corresponding to the different heights between the arm and the external ground.

In our simulations under the conditions of different heights to the ground, the distance of transmission was set as 20 cm, while the simulation frequencies were set as 3 MHz and 10MHz, which were corresponding to the cases that *R_b_*_11_ = 60.9 Ω, *C_b_*_11_ = 116 pF as well as *R_b_*_11_ = 54 Ω, *C_b_*_11_ = 33 pF, respectively. The corresponding simulation results are shown in Figure 12. According to Figure 12, the signal attenuation basically decreases with the increase of the height, in which the decrease of attenuation corresponding to 3 MHz is 1.39 dB in the case that the height increases from 1 cm to 30 cm, while the value corresponding to 10 MHz is 1.43 dB.

This phenomenon can be interpreted as that the capacitive coupling between the arm and the external ground becomes smaller as the height of the arm increases. For example, *C_g_*_1_ is equal to 22.83 pF when the height is 1 cm, while it is equal to 9.07 pF when the height is increased to 30 cm. Therefore, when the height decreases to some extent (such as less than 10 cm), the comparatively higher signal power is lost to the external ground through the capacitance betwe nen the arm and the ground, which leads to the comparatively bigger increase of signal attenuation. On the other hand, the attenuation becomes basically stable when the height is higher than 30 cm. For instance, the difference between the signal attenuation corresponding to 30 cm and the signal attenuation corresponding to 100 cm is only 0.12 dB when the frequency is 10 MHz. A similar phenomenon can be found in the results corresponding to 3 MHz. The main reason for this phenomenon can be interpreted as that the additional capacitance to the ground (*C_P_arm_*) decreases gradually with the increase of the height and finally reduces to zero, which is expressed as Equation (17).

## 5. Conclusions

In this paper, we propose an implant intra-body communication (IBC) method based on capacitive coupling, and investigate its transfer function and characteristics. Firstly, we derived the transfer function of the implant IBC based on capacitive coupling. Secondly, the corresponding parameters used in the transfer function were discussed. Finally, both the measurements of the proposed method and the corresponding simulations based on the transfer function were carried out under different conditions.

From the measurement and simulation results, we find that: (1) The simulation results based on the developed transfer function basically coincide with the measurements; (2) Compared with the implant IBC based on galvanic coupling, the proposed method has comparatively lower signal attenuation and basically stable frequency response within the frequency range of 2 MHz–40 MHz; (3) In the proposed method, the signal transmission distance almost has no influence on the signal attenuation; (4) The signal attenuation of the proposed method decreases with the increase of the height between body and the ground, and it becomes basically stable when the height is higher than a certain value, such as 30 cm. The above conclusions indicate that the proposed method of the implant IBC based on capacitive coupling has the advantages of low signal attenuation, insensitivity to signal transmission distance and so on. It will help to achieve an implant communication method for e-healthcare or u-healthcare with the characteristics of low power consumption and high transmission quality, *etc*.

## Figures and Tables

**Table 1.** The conductivity and relative permittivity of the materials.

**Materials**	**Physiological Saline**		**Insulating Shell**	
Parameters	*σ* (S/m)	*ε_r_*	*σ* (S/m)	*ε_r_*
Values	1.75	80.4	1 × 10^−14^	3
